# Broadening the definition of the genus *Thalassaphorura* Bagnall, 1949 (Collembola, Onychiuridae) with a new aberrant species from China

**DOI:** 10.3897/zookeys.364.6332

**Published:** 2013-12-17

**Authors:** Xin Sun, Louis Deharveng, Donghui Wu

**Affiliations:** 1 Key laboratory of Wetland Ecology and Environment, Northeast Institute of Geography and Agroecology, Chinese Academy of Sciences, Changchun 130102, China; 2 Muséum National d’Histoire Naturelle, UMR7205 du CNRS, CP50, 45 rue Buffon, 75005 Paris, France

**Keywords:** Thalassaphorurini, head chaetotaxy, tibiotarsi, labium

## Abstract

A new species belonging to the tribe Thalassaphorurini, *Thalassaphorura problematica* **sp. n.**, is described from Northeast China. The new species is closest to the large genus *Thalassaphorura* by its simple vesicles in PAO and its furcal rudiment, but it does not fit the definition of the genus by the absence of chaeta d0 on head, the number of chaetae in the distal whorl of tibiotarsi and the labium type. We discuss the relative weakness of these last characters at generic level, which lead us to assign the new species to *Thalassaphorura* instead of erecting a new genus. The diagnosis of *Thalassaphorura* is broadened accordingly.

## Introduction

The tribe Thalassaphorurini was established by Pomorski (1998), characterized by a furcal rudiment in a form of a finely granulated area with four small chaetae in two rows posteriorly. During recent investigations on Collembola in Northeast China, we recorded seven species belonging to three genera of the tribe Thalassaphorurini: *Allonychiurus* (*Allonychiurus songi* Sun & Wu, 2012), *Sensillonychiurus* (*Sensillonychiurus changchunensis* Sun & Wu, 2012, *Sensillonychiurus pseudoreducta* Sun & Wu, 2012, *Sensillonychiurus reducta* Sun & Wu, 2012), and *Thalassaphorura* (*Thalassaphorura encarpata* (Denis, 1931), *Thalassaphorura lifouensis* (Thibaud & Weiner, 1997), *Thalassaphorura macrospinata* Sun & Wu, 2012). Meanwhile, we also found a new species of the tribe Thalassaphorurini, with a combination of morphological characters that did not fit any of the known genera of the tribe. In the present paper, we assign the new species to the genus *Thalassaphorura* according to its simple vesicles in PAO and its furcal rudiment, rather than erecting a new genus. We broaden accordingly the diagnosis of *Thalassaphorura* and discuss the inconsistent characters of the new species. An updated key to the genera of the tribe Thalassaphorurini is provided.

## Material and methods

Specimens were collected by Berlese extraction of forest soil and humus, cleared in lactic acid and then mounted in Marc André II solution. They were studied using a Nikon Eclipse 80i microscope. The material is deposited in the Key Laboratory of Wetland Ecology and Environment, Northeast Institute of Geography and Agroecology, Chinese Academy of Sciences, Changchun.

Labial types are named after Fjellberg (1999). Labium areas and chaetal nomenclature follow Massoud (1967) and D’Haese (2003). Chaetae on anal valves are named after Yoshii (1996). Chaetae on the furcal area are classified in accordance with Weiner (1996). Tibiotarsus chaetotaxy formula follows Deharveng (1983), and is expressed as: total number of chaetae (number of chaetae in row C, number of chaetae in row B, number of chaetae in row A+T), for example 14 (1, 7, 6).

### Abbreviations used in descriptions

Ant.—antennal segments, PAO—postantennal organ, Th.—thoracic segments, Abd.—abdominal segments, ms—microsensillum, pso—pseudocellus, psx—parapseudocellus, Sp—posterior S-chaeta on Abd. V tergum, m—unpaired pseudopore or parapseudocellus.

The pseudocelli, parapseudocelli and pseudopores formula are the number of pseudocelli, parapseudocelli or pseudopores by half-tergum (dorsally) or half-sternum (ventrally) as follows: head anterior, head posterior/Th. I, Th. II, Th. III/Abd. I, Abd. II, Abd. III, Abd. IV, Abd. V (for instance: 32/133/33343).

## Systematics

### Family Onychiuridae Börner, 1913
Genus *Thalassaphorura* Bagnall, 1949

#### Thalassaphorura problematica sp. n.

http://zoobank.org/17661043-729B-4277-827D-E4F25F48FBC9

http://species-id.net/wiki/Thalassaphorura_problematica

##### Type material.

Holotype female; paratypes 9 females and 3 males on slides—China, Heilongjiang: Wulindong town, 46°33'N, 133°40'E, 16 Aug 2010, forest soil and humus, Wu Donghui, Liu Dong, Yuan Xiaoqiang and Yuan Yabin leg.

##### Diagnosis.

Pso formula as 32/133/33343 dorsally, 11/000/00010 ventrally; psx formula as 0/000/112001+1^m^ ventrally; Ant. III sensory organ with two granulated clubs (inner one bigger than outer); labium with 5 proximal chaetae; labial type AB; tibiotarsi of legs I–III with 14 (1, 7, 6) chaetae each; male ventral organ present on ventral tube as modified distal chaetae; anal spines 1.1–1.2 times as long as inner edge of hind unguis.

##### Description.

Body white in alcohol. Size 1000-1300 µm in females, 800–1100 µm in male; holotype: 1050 µm. Body subcylindrical, body sides parallel.

**Figure 1.**
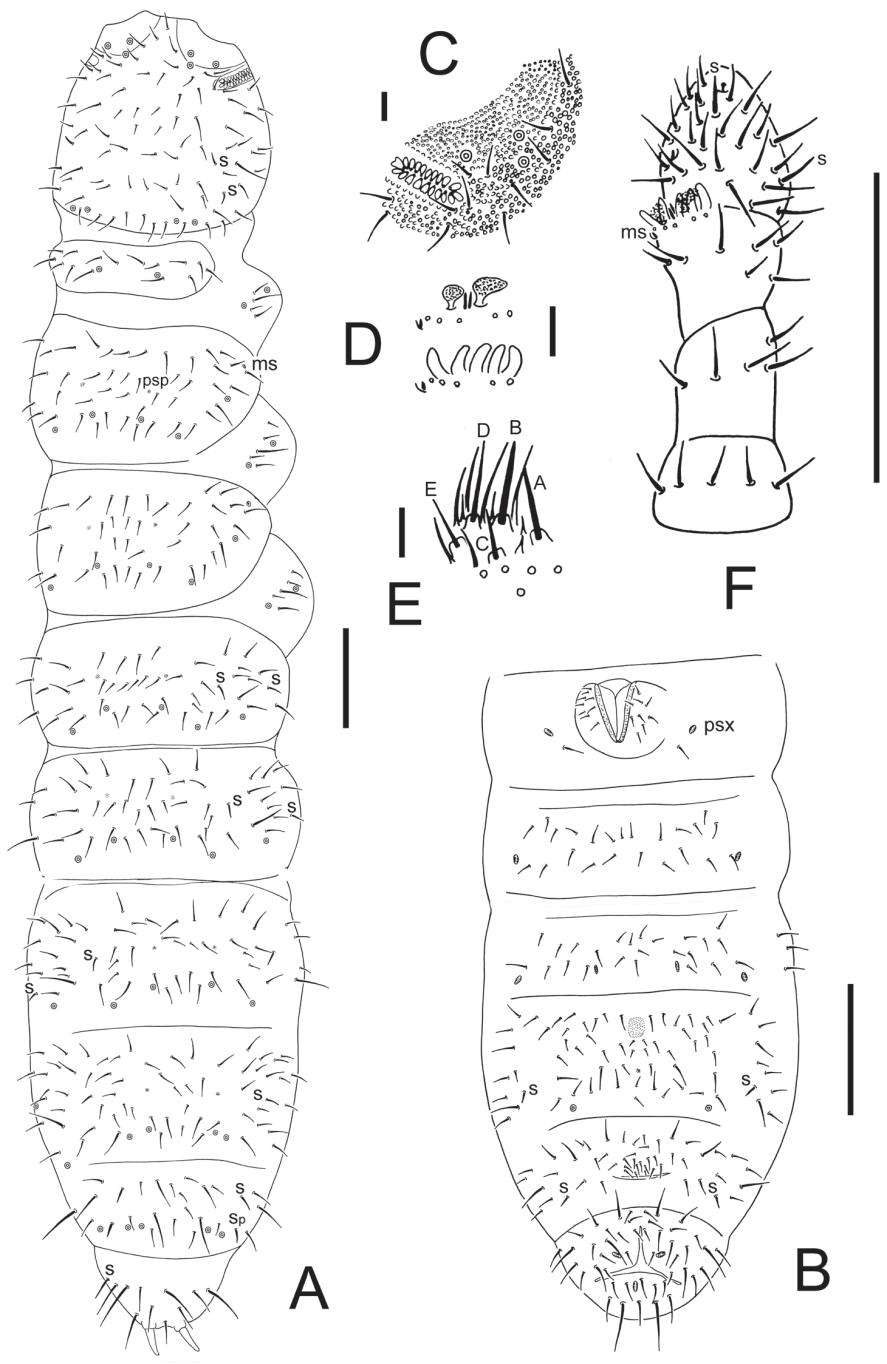
*Thalassaphorura problematica* sp. n. **A** dorsal side of body **B** ventral side of Abd. I–VI **C** PAO **D** clubs and papillae of AIIIO **E** Labium **F** Antenna. Scales: 0.1 mm (**A–B**, **F**), 0.01 mm (**C–E**).

**Figure 2.**
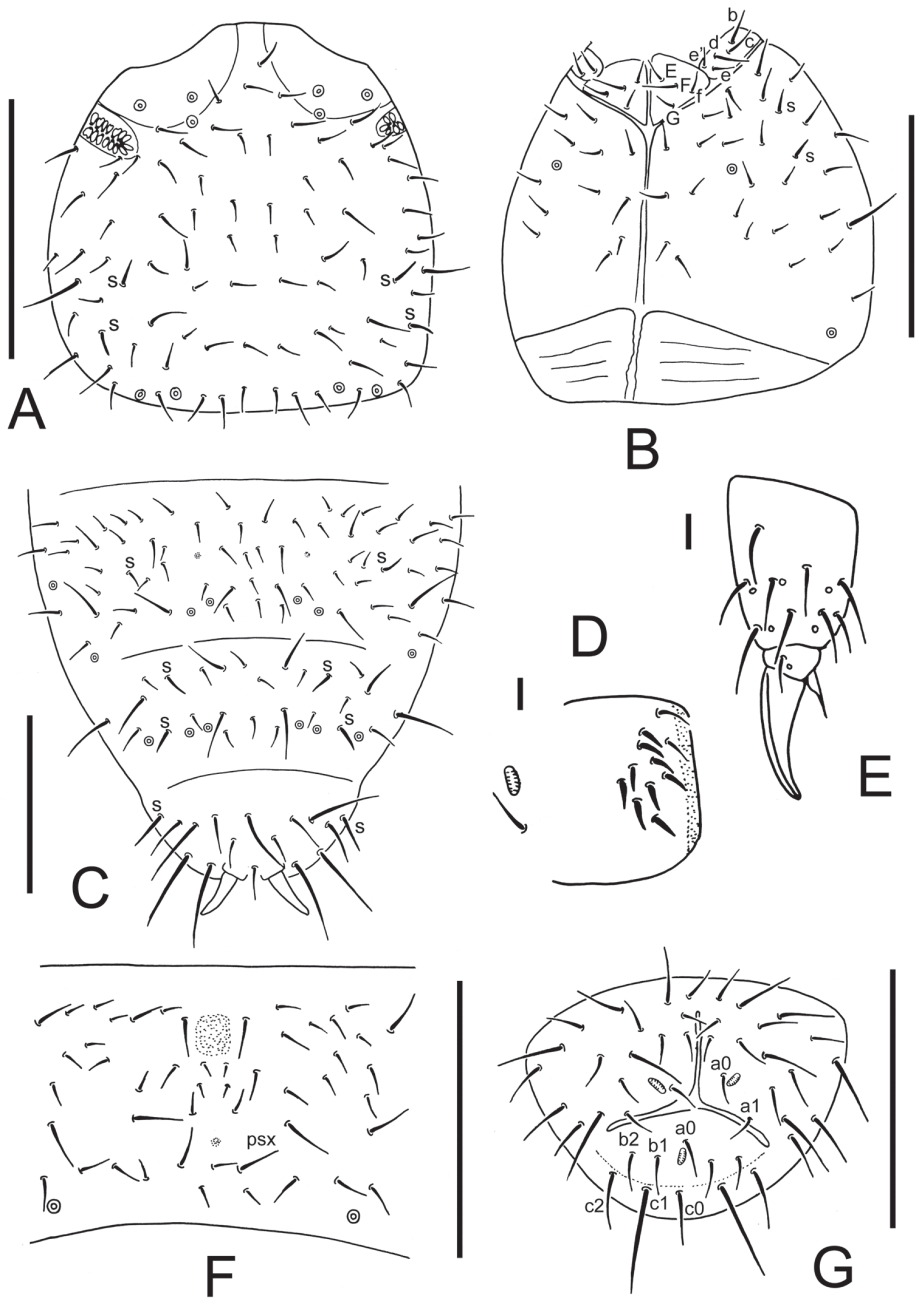
*Thalassaphorura problematica* sp. n. **A** dorsal side of head **B** ventral side of head **C** Abd. IV–VI terga **D** ventral tube (showing male ventral organ) **E** distal part of leg III **F** furca **G** anal valves. Scales: 0.1 mm (**A–C** and **F–G**), 0.01 mm (**D–E**)

Pseudocellar formula: 32/133/33343 dorsally, 11/000/00010 ventrally, subcoxa 1 of legs I-III with 2, 2 and 2 pso respectively (Fig. 1A, B). Parapseudocellar formula: 0/000/112001+1^m^ (each of anal valve with one psx) ventrally, absent dorsally (Figs 1A, B, 2G). Pseudopore formula: 0/011/11110 dorsally, 00/111/0001^m^0 ventrally (Fig. 1A, B).

Head. Antennae short and distinctly segmented, as long as head. Length ratio of Ant. I: II: III: IV as about 1: 1.5: 1.5: 1.5. Subapical organite of Ant.IV with globular apex; basolateral ms at about 1/3 length from base, above the second proximal row of chaetae (Fig. 1F). Ant. III sensory organ composed of 5 papillae, 5 guard chaetae, 2 sensory rods and 2 granulated clubs, the inner bigger than the outer, and a lateral ms (Figs 1D, F). Ant. II with 13 chaetae. Ant. I with 8 chaetae. Antennal base well marked. PAO composed of 20–24 simple vesicles (Fig. 1C). Dorsal cephalic chaeta d0 absent (Figs 1A, 2A). 3+3 p-chaetae present between two inner posterior pso, p1 anterior to others. Mandible with strong molar plate and 4 apical teeth. Maxilla bearing 3 teeth and 6 lamellae. Maxillary palp simple with 1 basal chaeta and 2 sublobal hairs. Labral formula 4/1,4,2;. Labium with 5 proximal, 4 basomedian (E, F, G, f) and 5 basolateral (b, c, d, e, e’) chaetae (Fig. 2B); labial type AB, papillae A–E respectively with 1, 4, 0, 3 and 2 guard chaetae (Fig. 1E). Head ventrally with 4+4 postlabial chaetae along ventral groove (Fig. 2B).

Body chaetotaxy. S-chaetae subcylindrical, apically rounded, 11/011/222121 dorsally, 11/000/000110 ventrally (Figs 1A, B); subcoxae 2 of legs I, II and III with 0, 0, 1 S-chaeta respectively. Tiny and blunt ms, present on Th. II–III. Ordinary chaetae differentiated into meso- and macrochaetae, ratio Sp: m1: p1 on Abd. V tergum = 1: 2–2.3: 0.8. Th. I tergum with 7–8+7–8 dorsal chaetae. Th. II–III terga with 4+4 chaetae and Abd. I–III terga with 3+3 chaetae along axis respectively (Fig. 1A). Abd. IV–V terga with one axial chaeta (p0) each, sometimes with asymmetric chaetae along axis. Abd. VI tergum with two axial chaetae (a0 and p0) (Figs 1A, 2C). Sterna of Th. I, II, and III with 0+0, 1+1, 1+1 chaetae respectively.

Appendages. Subcoxa 1 of legs I–III with 4, 5 and 5 chaetae, subcoxa 2 with 0, 4 and 4 chaetae respectively. Tibiotarsi of legs I, II and III with 14 (1, 7, 6) chaetae each (Fig. 2E). Unguis without teeth. Unguiculus short, about 0.3 times as long as inner edge of unguis, with inner basal lamella (Fig. 2E). Ventral tube with 1+1 basal and 8–11+8–11 distal chaetae (8–10+8–10 in female, 11+11 of which 9+9 modified in males) (Fig. 2D). Furca reduced to a field of fine granulation with 4 small dental chaetae arranged in 2 rows posteriorly; only one manubrial row of chaetae present posteriorly to dental chaetae (Fig. 2F).

Genital plate with 14–15 chaetae in females, 33–36 chaetae in male. Anal valves with numerous acuminate chaetae; each lateral valve with a0 and 2a1; upper valves with chaetae a0, 2b1, 2b2, c0, 2c1, 2c2 (Fig. 2G). Anal spines set on distinct papillae, 1.1–1.2 times as long as inner edge of hind unguis.

##### Derivatio nominis.

Named for its unusual characters among *Thalassaphorura*.

##### Discussion.

The new species is closest to the genus *Thalassaphorura* by its simple vesicles in PAO and the furcal rudiment. However, it does not match the definition of this genus proposed by Sun et al. (2010), nor those given previously by Weiner (1996), Fjellberg (1999) or Pomorski (1998) for three characters: absence of chaeta d0 on head, 6 chaetae in the distal whorl of tibiotarsi of all legs, and labium type AB. In order not to erect a new genus in a tribe in need of revision (Sun et al. 2011) and for a species otherwise very similar to existing *Thalassaphorura*, we placed our new species in the genus *Thalassaphorura* and broadened its diagnosis.

The new species belongs to the species-group of *Thalassaphorura* which has modified ventral chaetae in the adult male (“male ventral organ”), including the species *Thalassaphorura petiti* Sun & Wu, 2013, *Thalassaphorura bisetosa* Sun & Wu, 2013, *Thalassaphorura qinlingensis* Sun & Wu, 2013, *Thalassaphorura macrospinata* Sun & Wu, 2012 and *Thalassaphorura qixiaensis* Yan, Shi & Chen, 2006, all described from China. These species can be distinguished easily by the position or the number of modified chaetae of the male ventral organ, dorsal and ventral pso formula, and ventral psx formula.

Assigning the new species to this genus led us to re-examine three important taxonomic characters that separate the new species from most other *Thalassaphorura*.

The distal tibiotarsal chaetae have been recently checked in the genera *Allonychiurus*, *Onychiurus* and *Thalassaphorura* (Sun et al. 2010; Sun et al. 2011; Sun and Zhang 2012), showing that this character has a limited taxonomical value to discriminate these genera. In addition, paratypes of *Thalassaphorura petaloides* (Rusek, 1981) from Iraq and specimens of the same species from southern China were found to actually have 15 (1, 7, 7), 14 (1, 7, 6) and 14 (1, 7, 6)) chaetae on tibiotarsi I, II and III. Together with reduced tibiotarsal chaetotaxy of the new species described here, this leads us to extend the diagnosis of *Thalassaphorura* to species with 6, 7 or 9 chaetae in the distal row of tibiotarsus.

Chaeta d0 on head is considered as a stable character at the generic level. It is present in all species of *Thalassaphorura* (Sun et al. 2011) except *Thalassaphorura jailolonis* (Yoshii & Suhardjono, 1992) from Malukku (Indonesia) and the new species *Thalassaphorura problematica* sp. n. The species *Thalassaphorura jailolonis* was described in *Jailolaphorura* Yoshii & Suhardjono, 1992 (a subgenus of *Onychiurus*, upgraded to genus level by Weiner in 1996), but was subsequently transferred to *Thalassaphorura* by Bellinger et al. (1996–2013) according to a personal communication of Pomorski in 2002. This assignation is however uncertain because the chaetotaxy of the furcal rest is unknown in *Thalassaphorura jailolonis*. At this point, we consider that the diagnosis of the genus *Thalassaphorura* should provisionally state that d0 is present or absent on head, waiting for a re-examination of *Thalassaphorura jailolonis* on fresh material.

The third character, labium type, is not stable in several genera of Thalassaphorurini, being AC or ABC in *Allonychiurus* and *Sensillonychiurus* (Babenko et al. 2011), and even A, AC or ABC in *Thalassaphorura* (Sun et al. 2010). In our new species, labium is still of another type – AB. Moreover, labial type is undescribed in many species. This high intra-generic variability implies that this character should not be considered diagnostic at a generic level among Thalassaphorurini.

An amended diagnosis of the genus *Thalassaphorura* and an updated key of the genera of Thalassaphorurini integrating these changes are given below.

#### Thalassaphorura

Bagnall, 1949

http://species-id.net/wiki/Thalassaphorura

Onychiurus thalassophilus Type species: Bagnall, 1937

##### Diagnosis.

Postantennal organ oval, with numerous simple vesicles perpendicular to the long axis; antennal basis more or less indicated; clubs of AIIIO smooth, ribbed or granulated; Ant. IV with S-chaetae differentiated or not, ms close to the second row of chaetae, and no bulb on Ant. IV; labral chaetae formula 4/1,4,2; no multiplication of dorsal pseudocelli, 3 (rarely 4 or 2) pseudocelli in the antenno-basal group, 3–4 (rarely 2 or 5) pseudocelli per half-tergum on Abd. IV, 3 (rarely 4 or 2) pseudocelli per half-tergum on Abd. V (1–3 in a postero-internal group, one in a postero-lateral group); chaeta d0 on head present, rarely absent; Th. I usually with pseudocelli; Abd. VI with one or two axial chaetae (a0 or m0, or both); anal spines present or absent; distal whorl of tibiotarsal chaetae as 6, 7 or 9, no clavate tenent hairs; furcal rudiment as a finely granulated area with 4 small dental chaetae in two rows posteriorly, one manubrial row of chaetae present posteriorly to dental chaetae.

#### Key to genera of the tribe Thalassaphorurini

**Table:** 

1	Postantennal organ with simple vesicles	*Thalassaphorura*
–	Postantennal organ with compound vesicles	2
2(1)	Chaeta d0 on head present	3
–	Chaeta d0 on head absent	5
3(2)	Multiplication and unusual position of anterior pso on head and on Abd. IV–V	*Micronychiurus* Bagnall, 1949
–	Low number of dorsal pso in usual position	4
4(3)	Anal spines absent	*Agraphorura* Pomorski, 1998
–	Anal spines present	*Allonychiurus* Yoshii, 1995
5(2)	Distal whorl of tibiotarsi with 11 chaetae	6
–	Distal whorl of tibiotarsi with 7 or 9 chaetae	*Sensillonychiurus* Pomorski & Sveenkova, 2006
6(5)	Abd. V–VI terga fused, Abd. III sternum not divided in two sub-sterna	*Detriturus* Pomorski, 1998
–	Abd. V–VI terga not fused, Abd. III sternum divided in two sub-sterna	*Spinonychiurus* Weiner, 1996

#### Notes.

The genus *Thibaudichiurus* Weiner, 1996 was synonymized with the genus *Allonychiurus* by Babenko et al. (2011), but is still assigned to the tribe Thalassaphorurini by Bellinger et al. (1996–2013). Here we prefer to accept the synonym. The genus *Dungeraphorura*, of uncertain tribal position, has closer relation to the genera of the tribe Thalassaphorurini by three key characters, simple vesicles in postantennal organ, presence of d0 chaeta on head and 9 distal chaetae on tibiotarsi (Gulgenova and Potapov 2012). In the present key, we don’t include this genus because of its furcal rudiment, reduced to a cuticular pocket while it is reduced to a finely granulated area in the tribe.

## Supplementary Material

XML Treatment for Thalassaphorura problematica

XML Treatment for Thalassaphorura
